# Visual satisfaction with progressive addition lenses prescribed with novel foveal fixation axis measurements

**DOI:** 10.1038/s41598-023-38446-6

**Published:** 2023-07-12

**Authors:** Garcia-Espinilla Oscar, Sanchez Irene, Martin Raul

**Affiliations:** 1 Optometry Research Group, IOBA Eye Institute, School of Optometry, University of Valladolid, 47011 Valladolid, Spain; 2 Departamento de Física Teórica, Atómica y Óptica, Universidad de Valladolid, Paseo de Belén, 7 - Campus Miguel Delibes, 47011 Valladolid, Spain; 3 Instituto Universitario de Oftalmobiología Aplicada (IOBA), Universidad de Valladolid, Paseo de Belén, 17 - Campus Miguel Delibes, 47011 Valladolid, Spain

**Keywords:** Quality of life, Translational research

## Abstract

Progressive addition lens (PAL) prescription is usually conducted using the pupillary centre as a reference, which in general does not coincide with the visual axis (kappa distance), and this difference could induce undesired prismatic effects in far and near vision distances and adaptation problems. This study aimed to assess the impact on subjects’ visual satisfaction with PALs prescribed based on foveal fixation axis (FFA) measurements. Two different PALs (LifeStyle 3i, Hoya Lens Iberia) were randomly prescribed [one with a customized inset (the difference between the FFA measurements (Ergofocus®, Lentitech, Spain) at far and near distances and the second with a standard inset (2.5 mm)] to be used by 71 healthy presbyopic volunteers in a prospective double-masked crossover clinical study involving one month of use of each PAL. Patients were self-classified into four groups according to their previous experience with PALs: neophyte, PAL users, PAL drop-out, and uncomfortable PAL users. Visual function and overall satisfaction with each PAL were collected and compared. Ninety-seven percent (95% CI 93–100%) of participants successfully adapted to PALs prescribed with FFA without significant differences (*P* = 0.26) among the study groups (100% neophyte and uncomfortable PAL users (95% CI 100% in both groups), 89% (95% CI 67–100%) PAL users and 94% (95% CI 82–100%) PAL drop-out group). There were no statistically significant differences in visual function (*P* > 0.05) between customized and standard inset PALs. Customized and standard inset lenses showed similar satisfaction (*P* > 0.42) that increased significantly (*P* < 0.01 without any carry-over effect) after 30 days of wear. PALs prescribed with FFA measurements showed high visual satisfaction, suggesting that these measurements are suitable for prescribing PAL adaptation processes. Additional research is necessary to assess differences in PAL users’ performance with different prescription methods and lens designs.

## Introduction

Presbyopia is the age-related physiological and irreversible reduction in accommodative amplitude that induces symptoms of blur, discomfort and asthenopia to appear at near distances. Usually, it occurs during the fifth decade, and correction is required to achieve clear near visual acuity^1^. Currently, presbyopia affects 1 billion people globally^1^, and due to societal ageing, this number of people is expected to increase significantly by 2050^2^.

Several options are clinically available to correct presbyopia, such as contact lenses^3^, refractive surgery^1^, and pharmacological treatment^4^, but ophthalmic lenses are most often chosen, with progressive addition lenses (PALs) being the most popular option for most users and eye care practitioners^5,6^. PALs are one-piece spectacle design lenses with a progression of plus power across the lens surface from the distance prescription (the upper part of the lens) to the near prescription (the lower part of the lens, which is usually nasally decentred; this displacement distance is called the inset^7^). PAL lens design generates some lateral aberrations (especially Minkwitz astigmatism), which are explained by the Minkwitz theorem^8,9^. Lateral aberrations have a meaningful impact on the user’s vision^10,11^, and PAL users require an adaptation process with a variable duration between one and three weeks^5,12^. However, a certain percentage of users cannot tolerate vision with PAL lenses and need other forms of presbyopia correction^13^.

To improve the PAL adaptation process, accurate measurements of some facial parameters (nasopupillary, interpupillary distance and the fitting point height)^14^, usually measured with a frame ruler (Viktorin’s method^15^), several devices with limited agreement and repeatability^16,17^, and frame angles (pantoscopic and frame wrap)^18^ have been proposed. However, traditional facial measurements and PAL prescriptions are made using the centre of the pupil as a reference when it is well known that the pupil centre is not usually coincident with the eye visual axis (kappa distance^19^). To the best of the authors’ knowledge, only one device (Ergofocus®; Lentitech, Spain)^20^ has been proposed to measure the foveal fixation axis distance^21^ (FFA) to assist in prescribing PALs, because it provides FFA repeatable and non-interchangeable values with pupil-based measurements at both near and far distances.^20^.

The purpose of this clinical study was to assess subjects’ tolerance, visual satisfaction and visual function outcomes (central and lateral visual acuity, contrast sensitivity and stereopsis) with PALs prescribed based on FFA distances and the preference between standard (2.5 mm) and customized inset PALs in a sample of presbyopic participants classified according to their previous experience using PALs.

## Methodology

### Design

We used a randomized, double-masked crossover clinical study design. The sample size of 71 subjects was calculated based on the results of a previous pilot study, assuming a level of confidence of 95% (alpha error) and a statistical power of 95% (beta error) with a 10% withdrawal rate from the study.

### Subjects

Healthy, presbyopic participants from the University of Valladolid community were invited to participate in this study. All subjects must have a corrected far visual acuity of 20/30 or better. Patients younger than 40 years old or with severe systemic disease (multiple sclerosis, Parkinson’s disease, Alzheimer’s disease, cancer, myasthenia gravis and others), advanced glaucoma or corrected visual acuity under 20/30 were excluded from the study. Written informed consent was obtained from each subject after the Human Sciences Ethics Committee of Valladolid Area-Este Clinic Hospital (Castilla y Leon Public Health System-SACYL) approved the study (PI19-1194). All subjects were treated in accordance with the Declaration of Helsinki.

### Lens and frame prescription

To avoid the impact of the frame during the adaptation process (vertex distance, wrap and pantoscopic angles), each participant used the same frame throughout the study and at all study visits. To compensate for the facial differences among the participants, four similar unisex frames were available to choose the best frame for each participant at their first visit, two acetate frames and two metal frames, with eyesize ranged from 52 to 57 mm, bridge size from 17 to 19 mm and height from 33 to 39 mm. Each frame was selected according to subject’s facial morphological characteristics guaranteeing a proportionality of the parameters of the frame with subjects' physiognomy.

All participants were prescribed the same PAL lens design (LifeStyle 3i; Hoya Lens Iberia), randomizing the order of the use of PALs with a standard (2.5 mm) inset or with a customized inset (far and near FFA difference converted at the real frame vertex distance). Inset customization did not alter any other parameter of lens design to guarantee that differences just be related with inset differences. An independent researcher (I.S.) conducted the randomization and centred all PALs in the chosen frames.

### Study visits

The study protocol involved four visits, and two PALs were prescribed to be used by each participant over one month each in a random, masked order. An independent researcher (O.G-E.) conducted the clinical assessments of all participants.

At the first visit (inclusion visit), the participants received all of the study information, and informed consent was obtained prior to performing any clinical procedure. A complete eye exam was performed to ensure that patients met the inclusion criteria. Clinical data of far and near refraction, far LogMAR visual acuity, near Snellen visual acuity, far and near phoria (Maddox rod), near distance stereopsis (TNO test), and motor dominant eye (hole in the card test) were collected. Additionally, facial-FFA distances, fitting point height (frame ruler^17^), working and frame-vertex distance (frame ruler), pantoscopic and frame wrap angles (Essilor ruler^17^) distances were measured. The PAL lenses were then ordered from the manufacturer.

Participants were self-classified into four study groups based on their previous experience wearing PALs: neophytes (no previous experience wearing PALs), PAL users (previous and comfortable use of PALs), drop-out group (previous use of PALs and drop out due to discomfort), or uncomfortable PAL users (previous and uncomfortable use of PALs).

At the second visit (dispensing visit), the first spectacles with PAL lenses were double mask dispensed to wear for 1 month.

At the third visit (follow-up visit), visual function measurements with the first dispensed PAL spectacles were collected (straight forwards and 25° nasal and temporal lateral monocular and binocular visual acuity at far and near distances, contrast sensitivity (CSV 1000) and near distance stereopsis). After the clinical measurements were conducted, the second set of PAL lenses was dispensed in the same frame to be used during the next month.

Finally, at the fourth visit (final visit), the participants were assessed in the same way as in the third visit, and visual function measurements were collected while wearing the second PALs. After the clinical measurements, participants indicated in a multiple-choice questionnaire what spectacles they preferred with four options: none, both lenses, the first lenses or the second lenses.

In addition, to explore the adaptation process of each prescribed PAL during the second and third visits, all participants were instructed to fill out an online subjective questionnaire at two different times, the first on the tenth day of PAL use and the second after 1 month of PAL use. This questionnaire explores the participants’ general satisfaction with wearing the lenses with a single visual analogue scale question ranked between 1 and 10, with 1 indicating poor satisfaction and 10 indicating great satisfaction. Scores lower than 5 were considered as unsatisfactory PAL adaptation. All participants’ general satisfaction score (classified as satisfactory or unsatisfactory adaptation) were included in statistical analysis. Visual function records of participants unable to adapt to PALs were removed from the statistical analysis.

### FFA measurement

**Figure 1:**
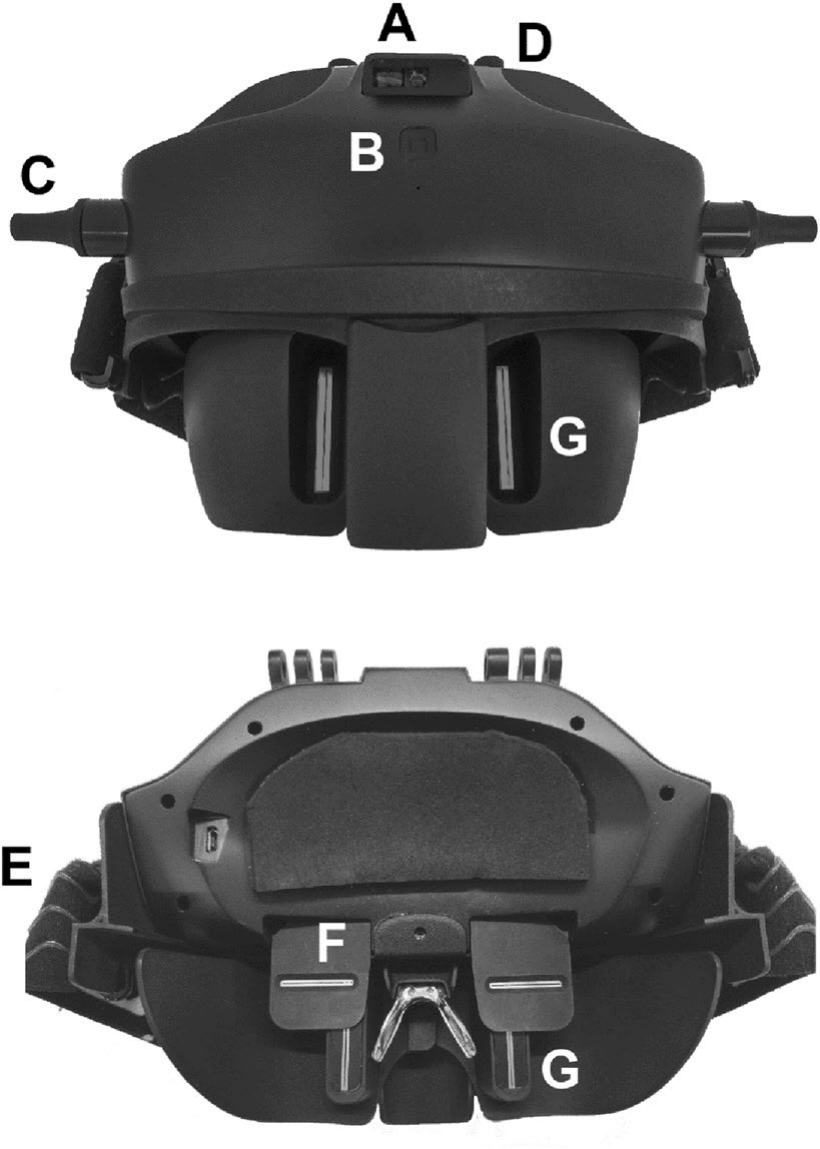
Front (upper) and back (inner) image of the Ergofocus® device designed for FFA distance measurement. (**A**) Distance sensor. (**B**) On/Off button. (**C**) Vertical slits displacement control. (**D**) Horizontal slits displacement control. (**E**) Rubber band. (**F**) Horizontal slit. (**G**) Vertical slit.

FFA distances were measured with the Ergofocus® system (Lentitech Inc., Spain) (Fig. 1) following a previously described procedure^20^. The patient wore the device on their head, fixed in place with rubber bands and resting on their nose. This device comprises two moveable stenopeic slits located in front of each eye (one horizontal and one vertical).

To measure the FFA distance in far vision, a fixation object was placed at 6 m, and to measure it in near vision, the fixation point was placed at a comfortable reading distance for the patient (both distances were measured and collected by the device’s sensor). First, the right eye FFA distance was measured. The slits were manually moved by the examiner until the patient could see the fixation object centred in their visual field and vice versa for the left eye. When the left eye measurement was completed, the examiner checked that the fixation point was centred binocularly. After the measurements, a file with the patient’s FFA measurement data was generated and saved in a tablet app via Bluetooth.

To measure right eye FFA measurement left eye was occluded, and right eye slits were manually moved by the examiner until the patient was able to see the fixation point centred in their visual field and the process was repeated in the left eye occluding the right eye. The same process was performed in the seated and supine position.

After each measurement, a file with the measurements is generated and saved in a tablet app via Bluetooth.

### Statistical analysis

Statistical analysis was performed using the SPSS 27.0 (SPSS, Chicago, IL, USA) statistical package for Mac. Normal distribution of the data was analysed using the Kolmogorov‒Smirnov test (*P* < 0.05 indicated that the data were non-normally distributed). Means, standard deviations (SDs), percentages and 95% confidence intervals (CIs) were used to describe the data where appropriate.

Differences among the study groups (neophytes, PAL users, drop-out users, or uncomfortable PAL users) were assessed with the Kruskal‒Wallis test. When differences were detected, a pairwise comparison with Bonferroni correction was used.

The percentage and 95% CI of PAL adaptation and lens preference were calculated with a 1000 sample boot-strap analysis, and the chi-square test was used to assess the significant differences in all samples and in each study group. To assess the impact of the order of wear of the prescribed PALs, the percentage of preference for first prescribed lenses was assessed with the chi-square test. Additionally, the carry-over effect of the satisfaction score with PALs at each study visit was analysed with a general linear model of repeated measures.

Differences in visual acuity, contrast sensitivity, stereopsis and satisfaction achieved with standard and variable inset PALs were assessed with the Wilcoxon nonparametric paired test, and differences among the study groups (neophytes, PAL users, drop-out users, or uncomfortable PAL users) were assessed with Kruskal‒Wallis test with Bonferroni correction when appropriate.

All statistical analyses were considered significant at *P* value < 0.05.

## Results

Seventy-one healthy subjects (37 women and 34 men) with a mean age of 54.01 ± 4.50 years (44 to 64 years) and a spherical equivalent of − 0.70 ± 2.52 D (− 7.50 to + 4.00 D) were included in this study. Forty-four of the patients had a right motor dominant eye, and 27 had a left motor dominant eye. These subjects were divided into four groups according to their previous use of PALs: 27 neophytes, 10 users adapted to PALs, 17 patients who previously dropped out of PAL use, and 17 users with an uncomfortable use of PALs. The descriptive results are summarized in Table 1.

**Table 1:** Summary of the descriptive values globally and divided by groups.

	Global (n = 71)	Neophyte (n = 27)	Comfortable PALs users (n = 10)	PALs drop out (n = 17)	Uncomfortable PALs user (n = 17)	*P*
Age (years)	54.01 ± 4.50	51.85 ± 3.34	56.20 ± 3.42	54.47 ± 3.71	55.71 ± 5.99	< 0.01†
	52.95 to 55.08	50.53 to 53.17	53.75 to 58.65	52.56 to 56.38	52.63 to 58.78	
Sex (W/M)	52% (40 to 63)	37% (19 to 56)	80% (50 to 100)	35% (12 to 59)	77% (59 to 94)	0.01‡
	/48% (37 to 60)	/63% (44 to 81)	/20% (0 to 50)	/65% (41 to 88)	/23% (6 to 41)	
Sphere RE (D)	− 0.34 ± 2.66	− 0.94 ± 2.30	1.45 ± 2.96	− 0.25 ± 2.92	− 0.53 ± 2.52	0.11
	− 0.97 to 0.29	− 1.84 to − 0.03	− 0.67 to 3.57	− 1.75 to 1.25	− 1.82 to 0.77	
Cylinder RE (D)	− 0.83 ± 0.81	− 0.70 ± 0.63	− 1.43 ± 1.57	− 0.83 ± 0.46	− 0.61 ± 0.32	0.48
	− 1.03 to − 0.62	− 0.97 to − 0.42	− 2.55 to − 0.30	− 1.09 to − 0.58	− 0.79 to − 0.42	
Sphere LE (D)	− 0.37 ± 2.40	− 0.64 ± 2.19	0.83 ± 2.65	− 0.51 ± 2.60	− 0.50 ± 2.38	0.34
	− 0.94 to 0.20	− 1.50 to 0.23	− 1.07 to 2.72	− 1.85 to 0.82	− 1.72 to 0.72	
Cylinder LE (D)	− 0.73 ± 0.57	− 0.72 ± 0.53	− 0.93 ± 0.85	− 0.72 ± 0.55	− 0.65 ± 0.44	0.93
	− 0.88 to − 0.59	− 0.94 to − 0.50	− 1.53 to − 0.32	− 1.02 to − 0.41	− 0.89 to − 0.41	
SE RE (D)	− 0.70 ± 2.62	− 1.23 ± 2.34	0.74 ± 2.78	− 0.62 ± 2.93	− 0.78 ± 2.52	0.13
	− 1.32 to − 0.08	− 2.16 to − 0.31	− 1.25 to 2.72	− 2.12 to 0.89	− 2.08 to 0.52	
SE LE (D)	− 0.70 ± 2.44	− 0.96 ± 2.28	0.36 ± 2.61	− 0.83 ± 2.72	− 0.79 ± 2.37	0.37
	− 1.28 to − 0.12	− 1.86 to − 0.06	− 1.50 to 2.23	− 2.23 to 0.57	− 2.00 to 0.43	
Addition (D)	2.12 ± 0.27	2.00 ± 0.26	2.20 ± 0.20	2.12 ± 0.20	2.25 ± 0.33	0.01†
	2.05 to 2.18	1.90 to 2.10	2.06 to 2.34	2.01 to 2.22	2.08 to 2.42	
RE VA (far)	− 0.03 ± 0.06	− 0.05 ± 0.06	− 0.02 ± 0.09	− 0.03 ± 0.04	0.01 ± 0.03	< 0.01†
	− 0.04 to − 0.01	− 0.07 to − 0.03	− 0.09 to − 0.05	− 0.05 to − 0.01	− 0.01 to 0.03	
LE VA (far)	− 0.03 ± 0.07	− 0.06 ± 0.06	− 0.03 ± 0.06	− 0.02 ± 0.06	0.02 ± 0.05	< 0.01†
	− 0.05 to − 0.01	− 0.09 to − 0.04	− 0.07 to 0.02	− 0.05 to 0.01	− 0.01 to 0.04	
RE VA (near)	1.00 ± 0.02	1.00 ± 0.00	1.00 ± 0.00	1.00 ± 0.00	1.01 ± 0.05	0.37
	1.00 to 1.01	1.00 to 1.00	1.00 to 1.00	1.00 to 1.00	0.99 to 1.03	
LE VA (near)	1.00 ± 0.02	1.00 ± 0.00	1.00 ± 0.00	1.00 ± 0.00	1.01 ± 0.05	0.37
	1.00 to 1.01	1.00 to 1.00	1.00 to 1.00	1.00 to 1.00	0.99 to 1.03	
Dominant eye (RE/LE)	62% (51 to 73)	74% (56 to 89)	30% (0 to 60)	65% (41 to 88)	59% (35 to 82)	0.11‡
	/38% (27 to 49)	/26% (11 to 44)	/70% (40 to 100)	/35% (12 to 59)	/41% (18 to 65)	
RE FFA (far)	29.56 ± 2.84	29.32 ± 2.45	28.63 ± 2.01	31.31 ± 3.54	28.72 ± 2.47	0.02†
	28.88 to 30.23	28.35 to 30.29	27.19 to 30.07	29.49 to 33.13	27.45 to 30.00	
LE FFA (far)	30.60 ± 3.41	30.30 ± 3.46	29.57 ± 3.16	31.48 ± 3.24	30.80 ± 3.70	0.52
	29.79 to 31.41	28.94 to 31.67	27.31 to 31.83	29.81 to 33.14	28.90 to 32.70	
RE INSET	1.37 ± 1.52	1.44 ± 1.38	1.81 ± 1.37	1.55 ± 1.34	0.85 ± 1.92	0.44
	1.02 to 1.74	0.89 to 1.99	0.83 to 2.79	0.85 to 2.24	0 to 1.84	
LE INSET	2.13 ± 1.84	2.77 ± 1.52	2.18 ± 1.47	1.60 ± 2.17	1.60 ± 1.95	0.09
	1.69 to 2.56	2.17 to 3.37	1.13 to 3.23	0.48 to 2.72	0.60 to 2.60	

FFA and INSET values are expressed in millimetres.

^†^ Pairwise comparison (Kruskal‒Wallis test with Bonferroni correction) showed that the neophyte group was younger (*P* = 0.03) than comfortable PAL users with lower addition (*P* = 0.01) and better far RE VA (*P* < 0.01) and LE VA (*P* < 0.01) than the uncomfortable PAL user group and that the PAL dropout group also showed higher far RE FFA distance (*P* = 0.03) than the uncomfortable PAL user group.

^‡^ Chi square test (X^2^_3_ = 11.54) for sex differences and (X^2^_3_ = 6.14) for dominant eye differences.

RE: right eye, LE: left eye, SE: spherical equivalent, VA: visual acuity, FFA: foveal fixation axis, n: sample size, PALs: progressive addition lenses, D: dioptre, W: women, M: men.

Data are presented as the mean ± standard deviation and 95% confidence interval. FFA and INSET values are expressed in millimetres.

No differences in FFA measurements were found between groups for the left eye (*P* = 0.52). However, for the right eye, differences were found between the drop-out and uncomfortable PAL user groups (*P* = 0.03). No differences were found among the other groups.

The customized inset measured in the right eye was significantly lower (*P* < 0.01) than the standard value (2.5 mm) in all study groups (neophyte group, *P* < 0.01; PAL drop-out group, *P* = 0.01; uncomfortable PAL users, *P* < 0.01) except in comfortable PAL users (*P* = 0.09). In the left eye, the uncomfortable PAL group also showed a significantly lower customized inset than the standard value (*P* = 0.02). No differences in the left eye between the customized and standard inset values were found in the neophyte group (*P* = 0.34), the comfortable PAL user group (*P* = 0.65) or the PAL drop-out group (P = 0.15).

Two participants dropped out of the study due to problems attending follow-up visits (mainly related to COVID-19 lockdown regulations). Ninety-seven percent (95% CI from 93 to 100%) of participants successfully used the prescribed PALs during all study visits, and only 3% (95% CI from 0 to 7%) (n = 2) reported an inability to adapt to both prescribed PALs (one was a previous PAL user, and the second participant was in the PAL drop-out group) (*P* < 0.01 Chi-square X^2^_1_ = 61.23).

Stratified by the study group, all (100%) neophyte and previously uncomfortable PAL user groups (95% CI of 100% in both groups) were able to adapt to PALs prescribed based on FFA distances, showing good far and near VA outcomes and PAL satisfaction. This percentage decreased slightly in the PAL drop-out group to 94% (95% CI from 82 to 100%) and in the previous PAL user group to 89% (95% CI from 67 to 100%) without statistically significant differences among the study groups (*P* = 0.26 Chi-square X^2^_3_ = 3.98).

### Lens preference analysis

**Figure 2:**
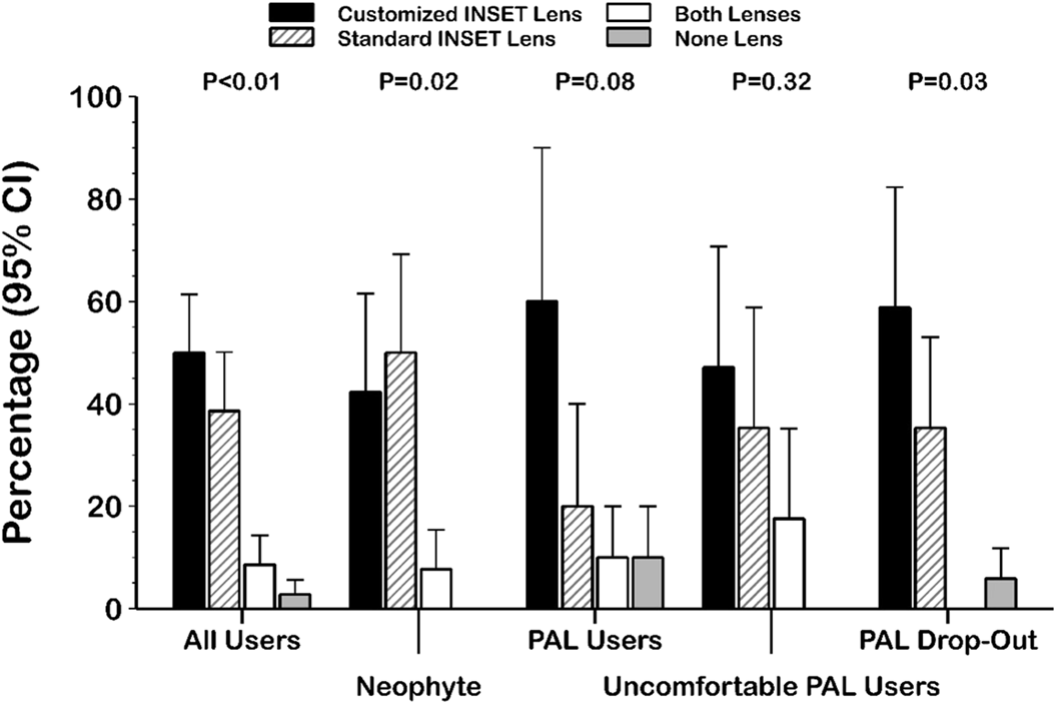
Summary of lens preference by group. Customized inset lens was preferred by 42% (95% CI from 23 to 61%) of neophyte users, 60% (95% CI from 30 to 90%) of PALs users, 59% (95% CI from 35 to 82%) of PALs drop-out users, and 47% (95% CI from 23 to 71%) of uncomfortable PALs users. PAL: progressive addition lens; CI: confidence interval.

Half of the participants preferred the customized inset lens, 50% (95% CI from 37 to 63%) compared with 39% (95% CI from 27 to 51%) of participants who chose the standard inset lens and a small number of participants, 8% (95% CI from 3 to 16%) without any preference; finally, just 3% (95% CI from 0 to 7%) of participants did not choose any lens (presented dissatisfaction with both lenses) (P < 0.01 Chi-square X^2^_2_ = 19.91). A detailed analysis of the study groups showed similar behaviour among the four groups studied (Fig. 2).

**Figure 3:**
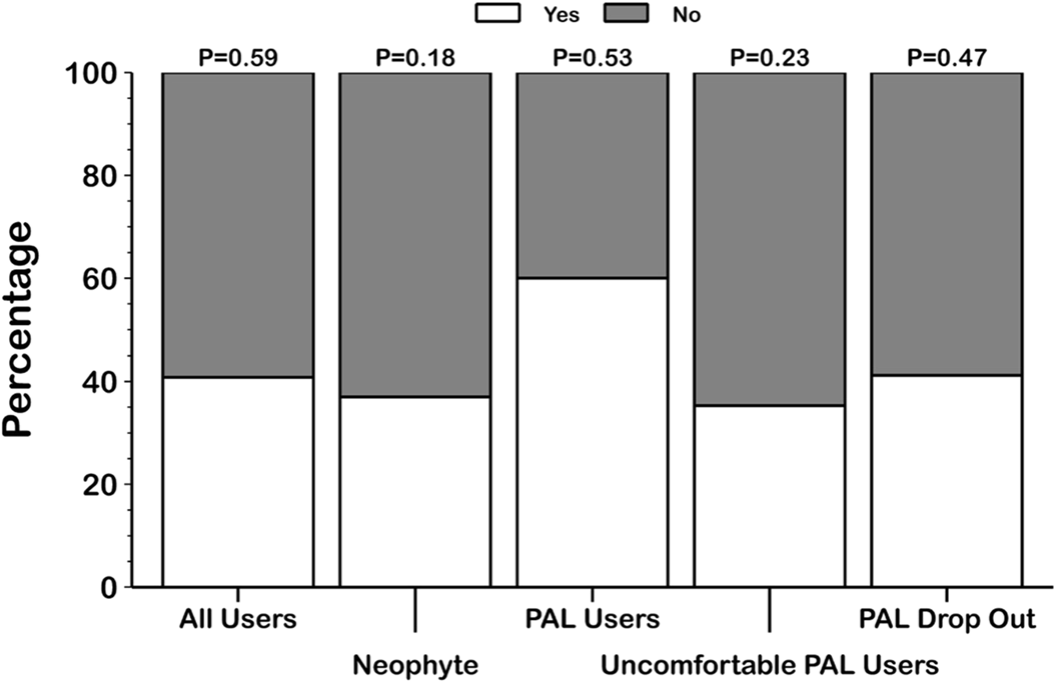
Details of the first prescribed lens preference. The first prescribed lens was chosen by 37% (95% CI from 18 to 55%) of neophyte users, 60% (95% CI from 30 to 90%) of PALs users, 41% (95% CI from 18 to 65%) of PALs drop-out subjects and 35% (95% CI from 12 to 59%) of uncomfortable PALs users at the end of the trial. PAL: progressive addition lens.

Preferences between the first and the second prescribed lenses was also analysed (Fig. 3). No statistical preference (*P* = 0.12 Chi-square X^2^_1_ = 2.38) was found; 41% (95% CI from 28 to 52%) of participants chose the first prescribed lens, and 59% (95% CI from 48 to 72%) chose the second prescribed lens. A similar trend was found in all study groups (*P* = 0.59 Chi-square X^2^_3_ = 1.90), where three groups (neophyte, PAL drop-out and uncomfortable PAL user) preferred the second prescribed lens (> 59%), but the PAL user group mostly chose the first prescribed lens (60%, 95% CI from 30 to 90%).

### Satisfaction analysis

**Figure 4:**
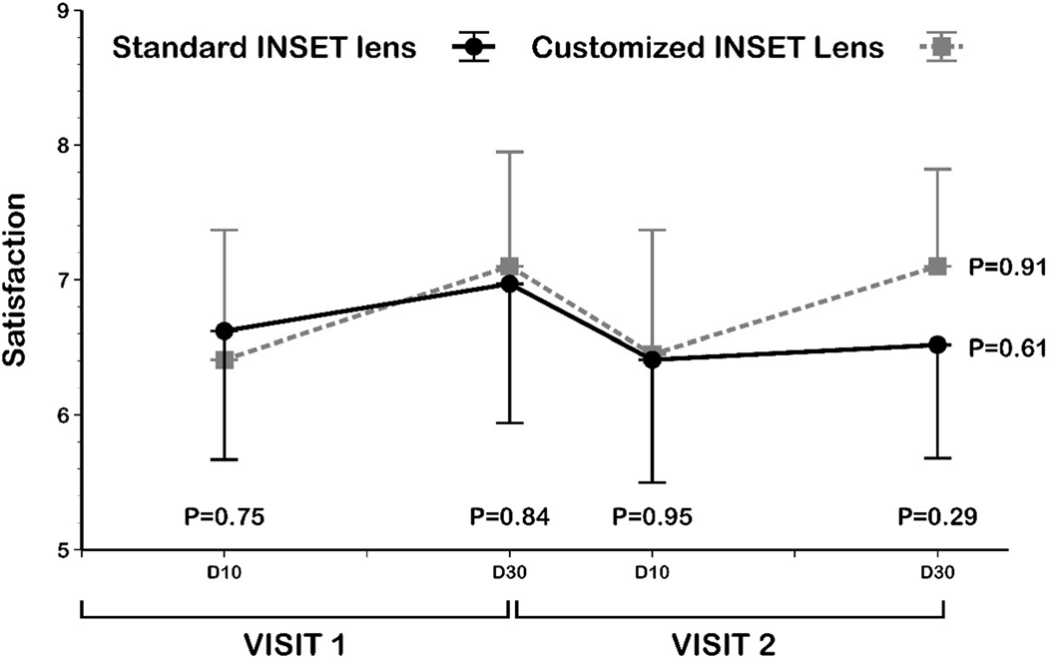
Comparison of the general satisfaction scale between the standard and customized inset lens and between the 10 and 30 days questionnaires. D10: day 10; D30: day 30.

No carry-over effect on the general satisfaction answers (Fig. 4) was found between visits and lenses, which allowed us to compare answers between the two PALs. All participants showed a statistically significant (*P* < 0.01 Wilcoxon rank test) satisfaction improvement with standard and customized inset lenses between Day 10 (6.56 ± 2.34 and 6.37 ± 2.41, respectively) and Day 30 (6.91 ± 2.24 and 7.06 ± 2.11, respectively). However, no satisfaction differences were found between customized and standard inset lenses on either survey day (*P* > 0.42 Wilcoxon rank test).

A detailed description of the satisfaction outcomes by each study group is provided in Table 2. All study groups, except PAL users with standard inset lenses, improved their satisfaction with prescribed PALs between days 10 and 30 (Table 2), but this change was only statistically significant (*P* < 0.01) in the neophyte group with customized inset lenses. The other groups did not show a statistically significant change in satisfaction between days (*P* > 0.06 Wilcoxon rank test) or PALs (*P* > 0.14 Wilcoxon rank test).

**Table 2:** Comparison of the general satisfaction scale between the standard and customized inset scales and between the Day 10 and Day 30 questionnaires.

Group	Lens	Day 10	Day 30	*P*	Percentage of change
Global (n = 71)	Standard IL	6.56 ± 2.34	6.91 ± 2.24	< 0.01	17.71 ± 86.34
	Mean ± SD (95% CI)	(6.01 to 7.12)	(6.38 to 7.45)		(− 3.03 to 38.45)
	Customized IL	6.37 ± 2.41	7.06 ± 2.11	< 0.01	32.75 ± 100.81
	Mean ± SD (95% CI)	(5.80 to 6.95)	(6.56 to 7.06)		(8.53 to 56.96)
	*P*	0.42	0.89	–	0.04
Neophyte (n = 27)	Standard IL	6.93 ± 2.30	7.19 ± 2.17	0.11	5.91 ± 13.88
	Mean ± SD (95% CI)	(6.02 to 7.84)	(6.33 to 8.04)		(0.41 to 11.40)
	Customized IL	6.44 ± 1.87	7.15 ± 1.89	< 0.01	17.15 ± 39.48
	Mean ± SD (95% CI)	(5.71 to 7.18)	(6.40 to 7.90)		(1.53 to 32.77)
	*P*	0.14	0.66	–	0.19
PALs users (n = 10)	Standard IL	6.90 ± 2.23	6.80 ± 2.90	0.79	− 2.46 ± 24.92
	Mean ± SD (95% CI)	(5.30 to 8.50)	(4.73 to 8.87)		(− 20.29 to 15.37)
	Customized IL	6.80 ± 2.62	7.30 ± 2.00	0.24	17.12 ± 34.48
	Mean ± SD (95% CI)	(4.93 to 8.67)	(5.87 to 8.73)		(− 7.54 to 41.79)
	*P*	0.73	0.81	–	0.07
PALs drop out (n = 17)	Standard IL	6.35 ± 2.69	7.12 ± 2.29	0.06	51.71 ± 169.02
	Mean ± SD (95% CI)	(4.97 to 7.74)	(5.94 to 8.29)		(− 35.19 to 138.62)
	Customized IL	6.06 ± 3.07	7.00 ± 2.34	0.09	73.88 ± 189.34
	Mean ± SD (95% CI)	(4.48 to 7.64)	(5.79 to 8.21)		(− 23.47 to 171.23)
	*P*	0.72	0.72	–	0.64
Uncomfortable PALs user (n = 17)	Standard IL	6.00 ± 2.15	6.31 ± 1.96	0.13	12.98 ± 27.86
	Mean ± SD (95% CI)	(4.89 to 7.11)	(5.27 to 7.36)		(− 1.86 to 27.83)
	Customized IL	6.31 ± 2.50	6.82 ± 2.40	0.09	18.92 ± 48.48
	Mean ± SD (95% CI)	(4.98 to 7.64)	(5.59 to 8.06)		(− 6.91 to 44.76)
	*P*	0.62	0.37	–	0.58

Mean ± standard deviation and 95% confidence intervals of satisfaction score (visual analogue scale). *P* value Wilcoxon rank test.

IL: inset lens, SD: standard deviation, CI: confidence interval, n: sample size, PALs: progressive addition lenses.

The percentage of change between Day 10 and Day 30 is described in Table 2. Customized inset PALs showed a significantly higher change than standard inset PALs (*P* = 0.04 Wilcoxon rank test). Analysis per study group revealed that the PAL drop-out group showed the largest percentage of change between Day 10 and Day 30 with customized and standard inset lenses (73.88 and 51.71%, respectively), without significant differences between lenses (*P* = 0.64). On the other hand, PAL users showed the lowest percentage of change with both lenses (17.12% with the customized inset lens and -2.46% with the standard inset lens).

### Visual function analysis

**Figure 5:**
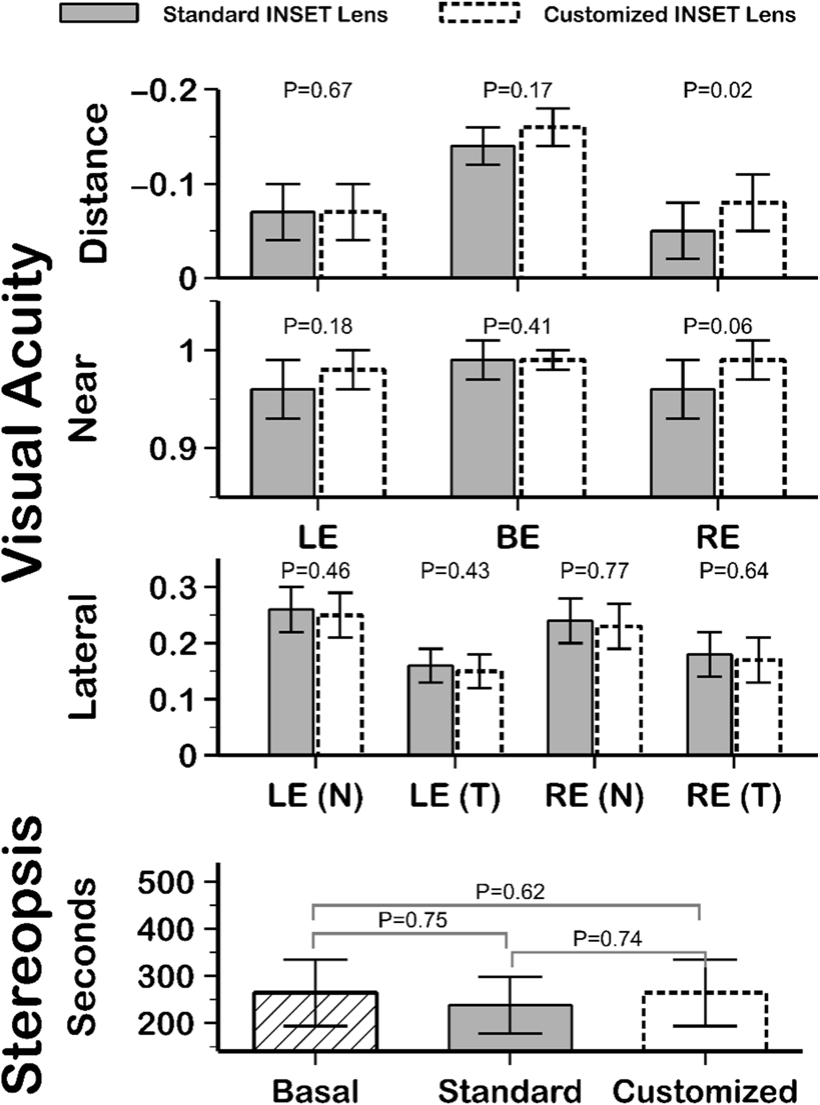
Summary of visual acuity (distance and near visual acuity and lateral visual acuity) and stereopsis measurements between standard and customized inset PALs. The Wilcoxon P value is presented. LE: left eye; RE: right eye; BE: both eyes; N: nasal; T: temporal.

**Figure 6:**
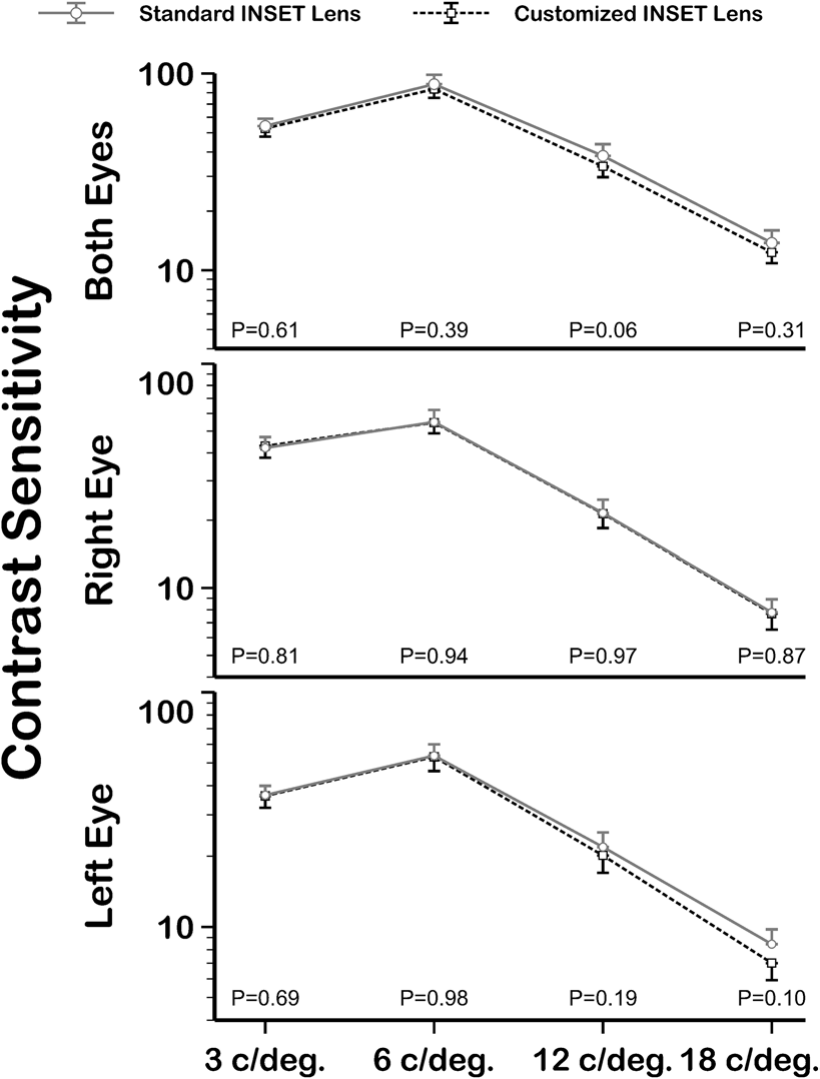
Summary of contrast sensitivity measurements between standard and customized inset PALs. The Wilcoxon *P* value is presented. c/deg = cycles/degree.

Differences between standard and customized inset PALs visual acuity (logMAR distance visual acuity and Snellen near and lateral visual acuity), stereopsis, and contrast sensitivity are summarized in Figs. 5 and 6 respectively. Customized inset lenses provided slightly better visual acuity at far and near distances than standard inset PALs, but the differences were statistically significant (*P* = 0.02) only in the right eye at far distances (Fig. 5 top). Any measurement of 25° lateral vision visual acuity showed statistically significant differences between both lenses (*P* > 0.4, Fig. 5 middle). The stereopsis achieved with both standard and customized inset PALs was also similar (*P* = 0.74 Wilcoxon rank test, Fig. 5 down). Finally, monocular and binocular contrast sensitivity outcomes were similar with both lenses (*P* > 0.06 Wilcoxon rank test) (Fig. 6). No carry-over effect was found for any of the visual function measurements during the study visits (*P* > 0.11).

## Discussion

Presbyopia is a visual condition that affects more people every year, with more than a billion people currently affected worldwide^22^. Furthermore, the quality of life of presbyopic patients is affected by PAL adaptation^22,23^. As PALs are the favourite option to correct presbyopia, it is important to guarantee and facilitate the best possible adaptation to improve the quality of life of these patients because PALs provide a better quality of life than single vision spectacles^23^. However, the correct centration of these ophthalmic lenses is essential to achieve good vision and user satisfaction^24^. Despite the importance of improving PAL adaptation and, consequently, presbyopic patients’ quality of life, surprisingly, there is a lack of studies assessing the influence of PAL centration on user satisfaction or proposing different procedures to improve the adaptation process to PAL use. Several PAL studies have reported the necessity of reliable measurements of facial parameters to achieve satisfactory adaptation^6,12,18,25,26^.

Some clinical studies have analysed clinical with PALs, comparing differences between customized, free-form, noncustomized and nonfree-form PALs.^6,18,27^ We found similar results that previous reports^18^ in visual function differences with different PAL, suggesting that standard clinical tests routinely performed to assess visual function would not reveal differences between different PAL use. This could mean that subjective preference is a subjects' decision based on their global experience after using the prescribed PAL that could include other situations that are difficult to measure in clinics with current techniques. Additionally, differences between PALs designed for computer use and regular PALs^12,28^ have been assessed by comparing users’ satisfaction and their subjective preference with different questionnaires^6,18,12,27,28^. Visual performance with PAL depends of the complex interrelation of several factors such as, optics of the lens, frame position, eye and head movement etc. and user individual characteristics, so it is usually assessed subjectively with questionnaires^6,18,12,27,28^. Nevertheless, there is no clinical study assessing clinical outcomes and users’ satisfaction with PALs dispensed with different pupillary centre-based facial measurement methods, with a lack of studies that assess repeatability, reproducibility, accuracy, or agreement between different methods to measure facial parameters to customize PAL prescriptions.

To the best of the authors’ knowledge, this is the first study to assess the clinical outcomes of a new method that uses FFA distance as a reference for prescribing PALs instead of traditional naso- or inter- pupillary distances. The results obtained in this study show that 97% of the PAL users were able to adapt satisfactorily to PALs prescribed based on the FFA distance. Furthermore, 100% of the previously uncomfortable PAL users and 94% of the PAL drop-out group were also able to comfortably use the prescribed PALs, indicating that the prescribed lenses improve their satisfaction compared with previously used PALs (prescribed with a pupillary centre centring method). These results suggest that using the FFA distance to prescribe PALs could allow users’ adaptation because the optical centre of the lens could be better aligned with the visual axes than the alignment achieved based on the pupillary centre or reflex.

The traditional and most popular method to clinically conduct pupillary measurements is Viktorin’s method with a frame ruler^15^. Nevertheless, this method has some limitations, highlighting a limited precision of 1 mm (which is the minimum unit of measurement), parallax errors^29^, and the misconception that this method does not consider the kappa distance. Kappa distance is the distance between the pupil centre or pupillary axes (the line from the centre of the entrance pupil that perpendicularly passes through the centre of the curvature of the cornea^21,30^) and the visual axis^19^ (the line connecting the fixation point with the foveola passing through the two nodal points of the eye^30^, which is simplified as a single point in some reports^21^) at the corneal plane^21,30^.

According to different reports, the kappa distance at the corneal plane has a value between 0.3 and 0.9 mm^31–34^ that will be higher at the frame plane [commonly located at 12 mm in front of the cornea (the vertex distance)]. Consequently, considering FFA instead of the pupil centre as the measurement reference could facilitate PAL users’ satisfaction and adaptation because a 97% PAL adaptation rate was achieved in this study, suggesting that the FFA distance could be an adequate method to prescribe PALs. However, further research comparing the adaptation rate after prescribing PALs based on traditional measurements (naso- or inter- pupillary distances) with those prescribed based on FFA measurements is necessary to assess which measurement would be preferable to facilitate users’ adaptation to PAL wear.

The inset value is an important PAL parameter to achieve satisfactory adaptation to PALs^7^ because a correct inset selection will place the near zone vision in a suitable position for the user’s convergence, allowing comfortable use in near vision. So, repeatable, and accurate measurements are necessary to allow inset PAL customization as FFA measurements have showed because Ergofocus® is the first method designed to measure FFA and inset specifically^20^. Unfortunately there is no accepted and validated method to calculate or measure the inset in PAL prescriptions^20^ and manufacturers use theoretical mathematical models and/or nonpublic algorithms or formulas to calculate this value. Therefore, low repeatability of pupillary based devices hinders proper inset customization in PAL prescription^16–18^. However, the satisfaction results achieved in this study suggest that the inset distance is not a key factor in PAL adaptation because no statistically significant user satisfaction difference was found between standard and customized inset PALs (Table 2). In fact, 39% of participants preferred the standard inset lens (Fig. 2), and no statistically significant differences were found between visual function achieved with standard or customized inset PALs (Figs. 5 and 6).

Additionally, it is common for manufacturers to use a standard value of 2.5 mm^7^. The FFA method assessed in this study allows the clinical measurement of the inset value, which is significantly different (*P* < 0.05) from the standard value of 2.5 mm for the right eye in all groups assessed except in the group of users previously satisfactorily adapted to PALs (*P* = 0.15). This result suggests that only subjects with real inset values similar to the standard 2.5 mm inset could easily adapt to standard inset PALs.

Surprisingly, nonstatistically significant differences were found in PAL users’ satisfaction between standard versus customized inset lenses, measured with a visual analogue scale. However, participants with customized inset PALs showed a significantly higher improvement in their satisfaction after three weeks of use (*P* = 0.04). It is noted that the PAL drop-out group showed the highest improvement in satisfaction between Day 10 and Day 30 of use (up to 74% of improvement), suggesting that users unable to adapt previously to PALs could benefit from the FFA measurement to prescribe PALs, especially with customized inset lenses.

This is a common pattern in PAL clinical studies that usually use the subjective preference of PAL users between two lenses measured with different subjective questionnaires as the main study parameter^6,18,12,27,28^. The subjective preference and user satisfaction found in this study suggest that the alignment between the FFA and the optical centre of the PALs could be a key factor in facilitating PAL adaptation, and other parameters, such as inset, lens design or lens corridor, could play a less relevant role in user satisfaction. Patients’ satisfaction is usually measured with VAS scale^35^ in vision and other health research, classifying as satisfied patients with a score higher than 5^36,37^ and highly satisfied patients with a score higher than 8 over 10^36,38^. However, there is not an accepted VAS score to classify subjects’ satisfaction.

### Prospects

This study is the first to analyse PAL user satisfaction with customized versus standard inset lenses prescribed with a new measurement method that considers FFA as the reference to centre PALs instead of the traditional methods based on pupillary measurements. Additional research will be necessary to compare users’ preference and satisfaction with PALs prescribed with FFA versus naso- or inter- pupillary distances and to assess the impact of the inset (customized versus theoretically calculated) and PAL designs (corridor distance; far, intermediate and near zone width; etc.) on users’ visual performance, satisfaction and adaptation to PALs to reduce the rate of patients unable to adapt to PALs and to improve presbyopia correction, patient management and patient quality of life. Additionally, FFA measurements could be used to prescribe other ophthalmic lenses, especially in high-power prescriptions.

### Study limitations

The main limitation of this study could be related to the small sample size and that it only included Caucasian subjects aged between 40 and 65 years. However, this sample size was calculated to achieve the necessary statistical power to conduct this study and a crossover design with adequate statistical analysis, including a carry-over assessment, followed the recommendations to assess crossover studies^39^ and guarantee the study conclusions.

PALs adapted with FFA were not directly compared with pupillary centre lenses because this comparison will require twice as many subjects and would have made it difficult to find enough volunteers, especially in the noncomfortable PAL user group. Moreover, in this study, only one PAL design (LifeStyle 3i, Hoya Lens Iberia) was used to avoid any impact of the PAL design on the study results, but it could be necessary to extend this analysis to other PAL designs to assess the impact of lens design on PAL user satisfaction and vision performance.

Although the participants were divided into four groups according to their previous use of PALs, it was not possible to assess the reason for their dissatisfaction among the uncomfortable and drop-out PAL users because in many cases, previous information on PAL design, frame, etc., were not available or identifiable.

To reduce the number of variables in this study, only standard and customized inset lenses were assessed because if additional parameters between lenses were included, a higher number of patients would need to be included. This fact has a limited impact on the study conclusions because the main study aim was to assess whether FFA allows PAL adaptation and to measure user satisfaction. Additional research will be necessary to assess or analyse other parameters affecting PAL users’ adaptation and satisfaction.

Another limitation of this study could be related with the impact of subjects’ age in study results, because it is expected higher PAL addition in older participants. So, further research assessing satisfaction and visual function outcomes according to PAL addition is necessary.

Finally, the absence of similar studies assessing or comparing PAL prescribing measurement methods made it challenging to compare our study results with previous studies. The main interest in previous reports of PALs was the subjective comparison of different types of lenses or designs, but there are no other studies that analysed nonpupillary-based methods to prescribe PALs.

## Conclusion

FFA measurements allow users to achieve a high visual satisfaction and adaptation rate to PALs prescribed for the correction of presbyopia. The presbyopic patients in this study, especially those with previous problems using PALs (uncomfortable use or previous PAL drop-out users), slightly preferred customized inset PALs. Additional research to assess the use of FFA measurements with other conditions and its application in PAL design, and to compare its clinical outcomes with other PAL-centring methods, is necessary.

## Data Availability

The datasets used and/or analysed during the current study available from the corresponding author on reasonable request.
